# *Timp3* Deficient Mice Show Resistance to Developing Breast Cancer

**DOI:** 10.1371/journal.pone.0120107

**Published:** 2015-03-25

**Authors:** Hartland W. Jackson, Carlo V. Hojilla, Ashley Weiss, Otto H. Sanchez, Geoffrey A. Wood, Rama Khokha

**Affiliations:** Ontario Cancer Institute, University of Toronto, Toronto, Ontario, Canada; Southern Illinois University School of Medicine, UNITED STATES

## Abstract

*Timp3* is commonly silenced in breast cancer, but mechanistic studies have identified both tumor promotion and suppression effects of this gene. We have taken a genetic approach to determine the impact of *Timp3* loss on two mouse models of breast cancer. Interestingly, MMTV-PyMT *Timp3^−⁄−^* mice have delayed tumor onset and 36% of MMTV-Neu *Timp3^−⁄−^* mice remain tumor free. TIMP3 is a regulator of TNF signaling and similar to *Timp3*, *Tnf* or *Tnfr1* loss delays early tumorigenesis. The tumor suppression in *Timp3* null mice requires *Tnfr1*, but does not result in alterations in the local immune compartment. In the mammary gland, *Timp*s are highly expressed in the stroma and through the transplantation of tumor cells we observe that *Timp3* deficiency in the host is sufficient to delay the growth of early, but not advanced tumor cells. Together our data is the first to identify a tumor promoting role of endogenous *Timp3 in vivo*, the spatial and temporal windows of this effect, and its dependence on *Tnfr1*.

## Introduction

Breast cancer is one of the most frequently diagnosed cancers and is the second leading cause of cancer deaths in women. The deletion and/or alteration of multiple signaling components are necessary in order to bypass the many protective functions a cell has for the prevention of cancer. In addition to genetic mutation, gene expression is often altered by DNA methylation or miRNA silencing during cancer progression. *TIMP3* is a common target of gene silencing in many cancer subtypes including colon, esophageal, brain, lung and breast cancers[1–3]. When the individual cell components of human breast cancer were investigated *TIMP3* was not expressed in normal epithelium or in ductal carcinoma in situ (DCIS), but was overexpressed in myofibroblasts[4]. The overexpression of *TIMP3* has both promoted the transformed phenotype[5] and caused cell death[6] of cancer cell lines. These contradictory studies have left the role of *TIMP3* in breast cancer undefined.

Classically the TIMPs (Tissue Inhibitors of Metalloproteinases) are considered tumor suppressors as they inhibit the degradation of structural components by matrix metalloproteinases (MMPs), a function necessary for both increased tumor invasion and angiogenesis. Among the TIMP family, TIMP3 is unique in that it is tightly bound to the extracellular matrix via heparan sulfate and it has the most broad protease-inhibition profile which in addition to MMPs includes many ADAMs (a disintegrin and metalloproteinase)[7,8]. Specifically, TIMP3 is the sole inhibitor of ADAM17, also known as tumor necrosis factor alpha (TNF) converting enzyme (TACE)[9]. TACE is an important sheddase that cleaves and activates growth factors important for EGFR signaling as well as inflammatory cytokines[10]. We have shown that through inhibition of TACE, TIMP3 regulates many functions of TNF signaling and inflammation[11–15]. It can activate this pathway through increased shedding and release of TNF, but it can also dampen its activation by shedding TNF receptors.

As a major inflammatory cytokine, TNF has a paradoxical, context dependent impact on cancer progression. It is known to be necessary for tumor induction in an inflammation dependent model of skin cancer, but has also been used as a cytotoxic agent against malignant cells[16,17]. TNF is produced both by malignant cells and the invading immune component of the tumor microenvironment[17].

Here, we determine the role of TIMP3 through TNF in different compartments and during different stages of a breast cancer mouse model that is independent of ErbB/EGFR growth factor release[18]. We find that in the absence of *Timp3* early breast cancer progression is delayed, but the growth of late stage carcinoma is accelerated. Further, we establish TNF signaling as an important regulator of early luminal breast cancer formation and identify the requirement of *Tnfr1* for mediating breast cancer suppression seen in the absence of *Timp3*.

## Results

### Loss of *Timp3* suppresses mammary tumorigenesis

To study the impact of *Timp3* in breast cancer development and progression we crossed *Timp3* ^*−⁄−*^ mice with MMTV-PyMT (PyMT) or MMTV-Neu (Neu) transgenic mice that represent well-accepted models of human breast cancers; all mice were on the pure FVB background. PyMT utilizes viral polyomal middle T oncogene, while Neu depends on ectopic mammary ErbB2 overexpression with mammary glands undergoing a multi-step tumorigenesis process. Typically, tumor palpation assesses tumor initiation, the period between onset and tumor endpoint reflects the rate of tumor progression, and these tumors culminate in spontaneous lung metastasis.

**Fig 1:**
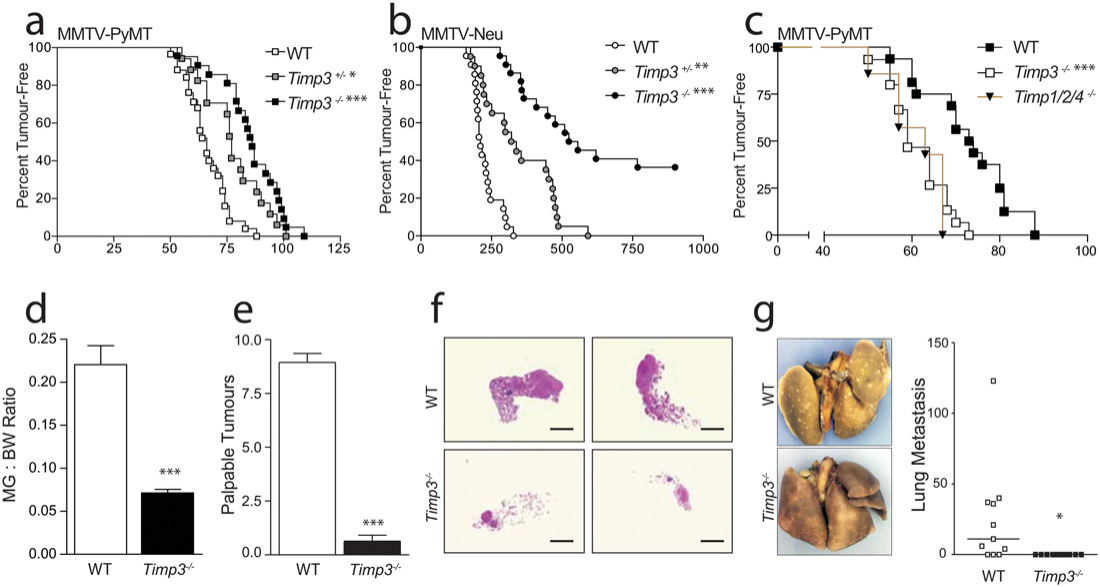
Loss of *Timp3* suppresses mammary tumorigenesis. **a**) Kaplan-Meier curve of the age at first detection of MMTV-PyMT positive *Timp3* ^*+/+*^ (n = 25), *Timp3* ^*+/-*^(n = 17), and *Timp3* ^*−⁄−*^ (n = 21) mammary tumors. **b**) Kaplan-Meier curve of the age at first detection of MMTV-Neu positive *Timp3* ^*+/+*^(n = 21), *Timp3* ^*+/−*^ (n = 20), and *Timp3* ^*−⁄−*^ (n = 22) mammary tumors. **c**) Kaplan-Meier curve of the age at first detection of MMTV-PyMT positive *Timp3* ^*+/+*^, *Timp3* ^*−⁄−*^, and *Timp1* ^*−⁄−*^ *Timp2* ^*−⁄−*^ *Timp4* ^*−⁄−*^ mammary tumors. Tumor burden at Day 80 depicted by mammary gland to body weight ratios (**d**, n = 17, mean ± s.e.m), the total number of palpable tumors (**e**, n = 17, mean ± s.e.m), representative images of mammary wholemounts (**f**, scale bar 5mm), and representative images of Bouin’s stained lungs with quantification of macroscopic metastasis (**g**, line = median). *p<0.05, **p>0.01, ***p<0.001.

In the aggressive PyMT model, we noted a remarkable delay of tumor initiation and metastasis in the *Timp3* null group. The median age of first detection was 66 days in *PyMT Timp3* ^*+/+*^ versus 86 days in *PyMT Timp3* ^*−⁄−*^ cohorts (Fig 1a). Comparison of mammary weights in 80-day-old mice reflected a marked reduction in tumor multiplicity and burden in *Timp3* null mice (Fig 1d and 1e), which was visualized by the overall decreased cellularity evident in wholemount staining (Fig 1f). Gross and microscopic assessment of lung metastases indicated multiple lesions in *PyMT Timp3* ^*+/+*^ tumor bearing 80-day-old mice, but these were completely absent in the *PyMT Timp3* ^*−⁄−*^ cohort (Fig 1g).

In parallel, we investigated the impact of *Timp3* loss on Her2-driven breast cancer, an oncogene overexpressed in ~25% of human breast cancer. We observed profound mammary tumor suppression in the *Timp3* deficient cohort (*Neu Timp3* ^*−⁄−*^) and decreased tumor incidence. Specifically, 36% of *Neu Timp3* ^*−⁄−*^ mice remained tumor free across their lifespan, even beyond >2 years of age (Fig 1b). Tumors were palpable in control Neu mice (*Neu Timp3* ^*+/+*^) at a mean age of 227 days, whereas in the *Neu Timp3* ^*−⁄−*^ mice that did develop tumors, onset was delayed to 453 days.

### Among *Timps*, only *Timp3* affects mammary tumorigenesis

We then examined whether *Timp3* heterozygosity impacts mammary tumor development in both models. Tumor initiation was delayed in *PyMT Timp3* ^*+/−*^and *Neu Timp3* ^*+/−*^ cohorts compared to their respective control groups (Fig 1a and 1b). This indicates haploinsufficiency whereby the loss of even one allele of *Timp3* is able to confer tumor protection. Altogether these data have led to the surprising finding that a stepwise loss of one or both alleles of *Timp3* delays or halts mammary cancer in two independent models of luminal breast cancer.

We next determined whether the modulation of breast cancer is a general function of the TIMP family, or if it is specific to *Timp3* status. There are 4 members in the mammalian *Timp* gene family. Triple knockout mice lacking the other three *Timps* (*Timp1* ^*−⁄−*^/*Timp2* ^*−⁄−*^/*Timp4* ^*−⁄−*^) were crossed into the PyMT model; all mice were in C57/Bl6 background. We found that MMTV-PyMT tumor initiation and progression was unaltered in compound mice lacking *Timp 1*, *2* and *4* (Fig 1c). These data identify the unique role of TIMP3 in breast cancer development.

### *Timp3* loss exerts suppression at an early stage of mammary tumorigenesis

**Fig 2:**
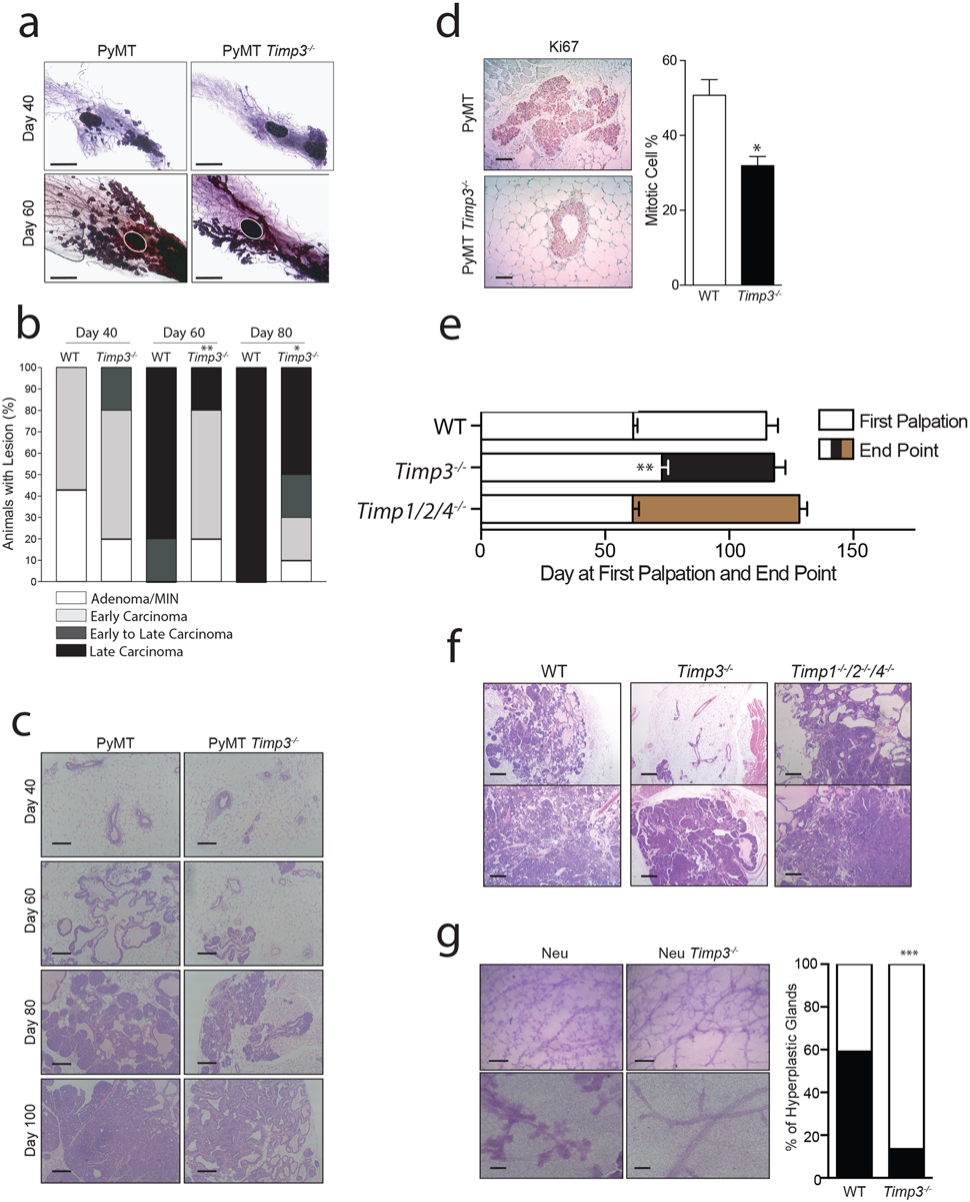
*Timp3* differentially impacts early versus late stages of mammary tumorigenesis. **a**) Wholemount images from Day 40 and 60 PyMT expressing glands; scale bar 1mm. Histological scoring of the most advanced lesion (**b**) as a percentage of all investigated *Timp3* ^*+/+*^ and *Timp3* ^*−⁄−*^ MMTV-PyMT tumors at Day 40, 60 and 80 with representative images (**c**, scale bar 500μm). **d**) Immunohistochemistry staining and quantification of the percent of Ki67 positive proliferating cells; scale bar 100μm. **e**) Histogram of the number of days to first palpation and from first palpation to tumor endpoint (mean ± s.e.m. *WT* n = 15, *Timp3* ^*−⁄−*^ n = 16, *Timp1* ^*−⁄−*^ *Timp2* ^*−⁄−*^ *Timp4* ^*−⁄−*^ n = 7; scale bar 500μm). **f**) Representative H&E images of endpoint MMTV-PyMT mammary tumors. **g**) Representative wholemount images of MMTV-Neu positive non-tumor bearing glands (scale bar 500μm top and 200μm bottom) and the percentage of investigated non-tumor bearing glands that are hyperplastic (*Neu Timp3* ^*+/+*^ n = 22, *Neu Timp3* ^*−⁄−*^ n = 37). *p<0.05, **p<0.01, ***p<0.001.

To better define the window of suppression in the *PyMT Timp3* ^*−⁄−*^ model, we performed further analyses at days 40, 60 and 80. First, visualization of mammary wholemounts revealed multiple tumor foci in *PyMT Timp3* ^*+/+*^ at day 40, which were absent in *PyMT Timp3* ^*−⁄−*^ glands (Fig 2a). These differences in cellularity were accentuated by day 60 where controls had many points of tumor initiation but *Timp3* ^*−⁄−*^ tumorigenesis was confined to specific ducts covering far less of the gland (Fig 2a).

The most advanced tumor or mammary lesion per gland was then scored histologically on days 40, 60 and 80. By the Annapolis classification, these tumors were designated as mammary intraepithelial neoplasia (MIN)/adenoma; early, early-late, or late carcinomas (Fig 2b and 2c)[19]. The control group (*PyMT Timp3* ^*+/+*^) tumors advanced from 40% MIN at day 40, to 80% late carcinomas at day 60, and 100% late carcinomas at day 80. The kinetics of tumor progression were decelerated in the *PyMT Timp3* ^*−⁄−*^ cohort. Although some aggressive lesions (early-late carcinomas) appeared as early as day 40, there were major differences at day 60 when controls had late carcinomas but knockout tissue predominantly still displayed early carcinomas. By day 80, the knockout glands continued to exhibit the full spectrum of lesions including MIN, whereas controls were 100% late carcinomas. Next, we stained mammary tumors for markers of proliferation (Ki67) and apoptosis (cleaved caspase-3). We observed very few cells undergoing apoptosis (data not shown) but a robust level of cellular proliferation. Ki67 positivity was lower in day 40 *PyMT Timp3* ^*−⁄−*^ tissue than controls (Fig 2d).

These mouse cohorts were followed through to humane tumor endpoints to determine the overall breast cancer survival. Despite delayed tumor initiation and decreased tumor numbers in *Timp3* ^*−⁄−*^ mice, the most advanced tumor reached experimental endpoint at the same time as in *PyMT Timp3* ^*+/+*^ mice (Fig 2e, S1 Fig). When examining the mammary glands of *PyMT Timp3* ^*+/+*^ end-point mice, we consistently noted that all glands were overcome by aggressive adenocarcinomas, but this was not the case in *PyMT Timp3* ^*−⁄−*^ glands that still contained areas of normal mammary duct (Fig 2f). Altogether, these data show the window of breast cancer suppression to be early during tumorigenesis, yet identify a small subset of tumors that are more aggressive in the absence of *Timp3* and able to progress rapidly to advanced disease.

With respect to the MMTV-Neu model, differences in tumor initiation were also observed. The Neu/Her2 oncogene first causes ductal hyperplasia before leading to full tumorigenesis. Wholemount staining of non-tumor-bearing glands from endpoint MMTV-Neu mice revealed that 13 of 22 Neu *Timp3* ^*+/+*^ glands were hyperplastic, while only 5 of 37 *Neu Timp3* ^*−⁄−*^ glands showed evidence of transformation (Fig 2g), further supporting this concept.

### *Timp3* requires *Tnfr1* to mediate mammary tumor suppression

**Fig 3:**
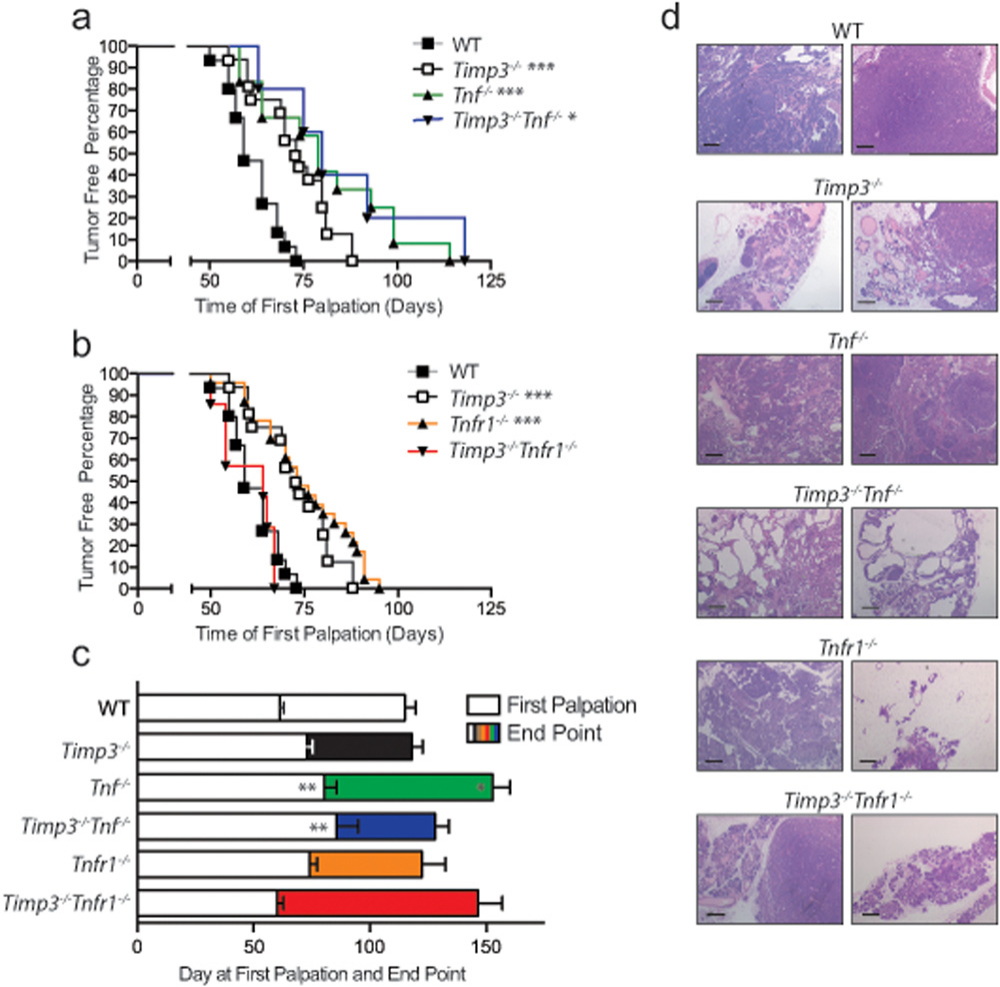
*Tnfr1* is required for tumor suppression in the absence of *Timp3*. **a**) Kaplan-Meier curve of the age at first detection of MMTV-PyMT positive WT (*Timp3* ^*+/+*^, n = 15), *Timp3* ^*−⁄−*^ (n = 16), *Tnf* ^*−⁄−*^ (n = 12) and *Timp3* ^*−⁄−*^ *Tnf* ^*−⁄−*^ (n = 5) mammary tumors. **b**) Kaplan-Meier curve of the age at first detection of MMTV-PyMT positive WT (*Timp3* ^*+/+*^), *Timp3* ^*−⁄−*^, *Tnfr1* ^*−⁄−*^ (n = 23) and *Timp3* ^*−⁄−*^ *Tnfr1* ^*−⁄−*^ (n = 7) mammary tumors. **c**) Histogram of the number of days to first palpation and from first palpation to tumor endpoint. **d**) Representative H&E images of endpoint MMTV-PyMT mammary tumors.

TIMP3 is a recognized regulator of TNF bioavailability, and TNF signaling has the capacity to either drive or suppress cancer progression[10,17]. To investigate whether the TIMP3 deficient mammary tumor phenotype involves TNF we undertook a genetic approach. We bred PyMT transgenic mice with *Tnf* ^*−⁄−*^ or *Tnfr1* ^*−⁄−*^ mice individually, or in combination with *Timp3* deficiency. *Tnf* deficiency on its own (*PyMT Tnf* ^*−⁄−*^) significantly delayed PyMT tumor initiation (first palpation in *PyMT Tnf* ^*−⁄−*^ mice occurred at Day 80 versus Day 61 in *PyMT Tnf* ^*+/+*^ mice, Fig 3a). We noted however that compound loss of *Timp3* and *Tnf* (*PyMT Timp3* ^*−⁄−*^ *Tnf* ^*−⁄−*^) did not further this suppression, suggesting overlapping functions of *Timp3* and *Tnf* during PyMT tumor initiation (Fig 3a). Comparison of tumor end points showed a ~25% extension of *PyMT Tnf* ^*−⁄−*^ lifespan over *Tnf* ^*+/+*^ (Fig 3c), whereas *PyMT Timp3* ^*−⁄−*^ *Tnf* ^*−⁄−*^ group exhibited intermediary survival.

The individual loss of *Tnfr1* (*PyMT Tnfr1* ^*−⁄−*^) also led to a longer time to tumor palpation, similar to *PyMT Timp3* ^*−⁄−*^ and *PyMT Tnf* ^*-/-*^ cohorts (Fig 3b). When combined with *Timp3* deficiency, *PyMT Timp3* ^*−⁄−*^ *Tnfr1* ^*−⁄−*^ mice have a distinct phenotype such that they failed to delay tumor initiation unlike all other *Timp3* ^*−⁄−*^, *Tnf* ^*−⁄−*^, or *Tnfr1* ^*−⁄−*^ cohorts. These data show that the effect of *Timp3* loss on mammary tumorigenesis depends upon *Tnfr1*. (Fig 3b). Tumor progression to end-point in *PyMT Tnfr1* ^*-/-*^ mice was also comparable to *PyMT Timp3* ^*−⁄−*^ and *PyMT Tnfr1* ^*−⁄−*^ cohorts. On the other hand, this phenotype was lost in compound *PyMT Timp3* ^*−⁄−*^ *Tnfr1* ^*−⁄−*^ mice, which exhibited the equivalent rapid tumor progression as in *PyMT* controls.

Histological examination was performed across all described cohorts. By their endpoint, delayed *PyMT Tnf* ^*−⁄−*^ tumors had completely advanced to aggressive carcinomas, but *PyMT Timp3* ^*−⁄−*^ *Tnf* ^*−⁄−*^ ductal structures had reached a critical tumor size partially through a large proportion of dilated hyperplastic ducts (Fig 3d). In contrast, PyMT *Tnfr1* ^*−⁄−*^ glands maintained the normal architecture of some of their ductal structures, like *PyMT Timp3* ^*−⁄−*^, but this characteristic was not apparent in compound *PyMT Timp3* ^*−⁄−*^ *Tnfr1* ^*−⁄−*^ mice (Fig 3d). These data provide further support to the proposed dependence on intact *Tnfr1* for mediating tumor suppression inherent to *Timp3* deficient glands.

### The loss of *Timp3* does not alter the mammary gland immune compartment

**Fig 4:**
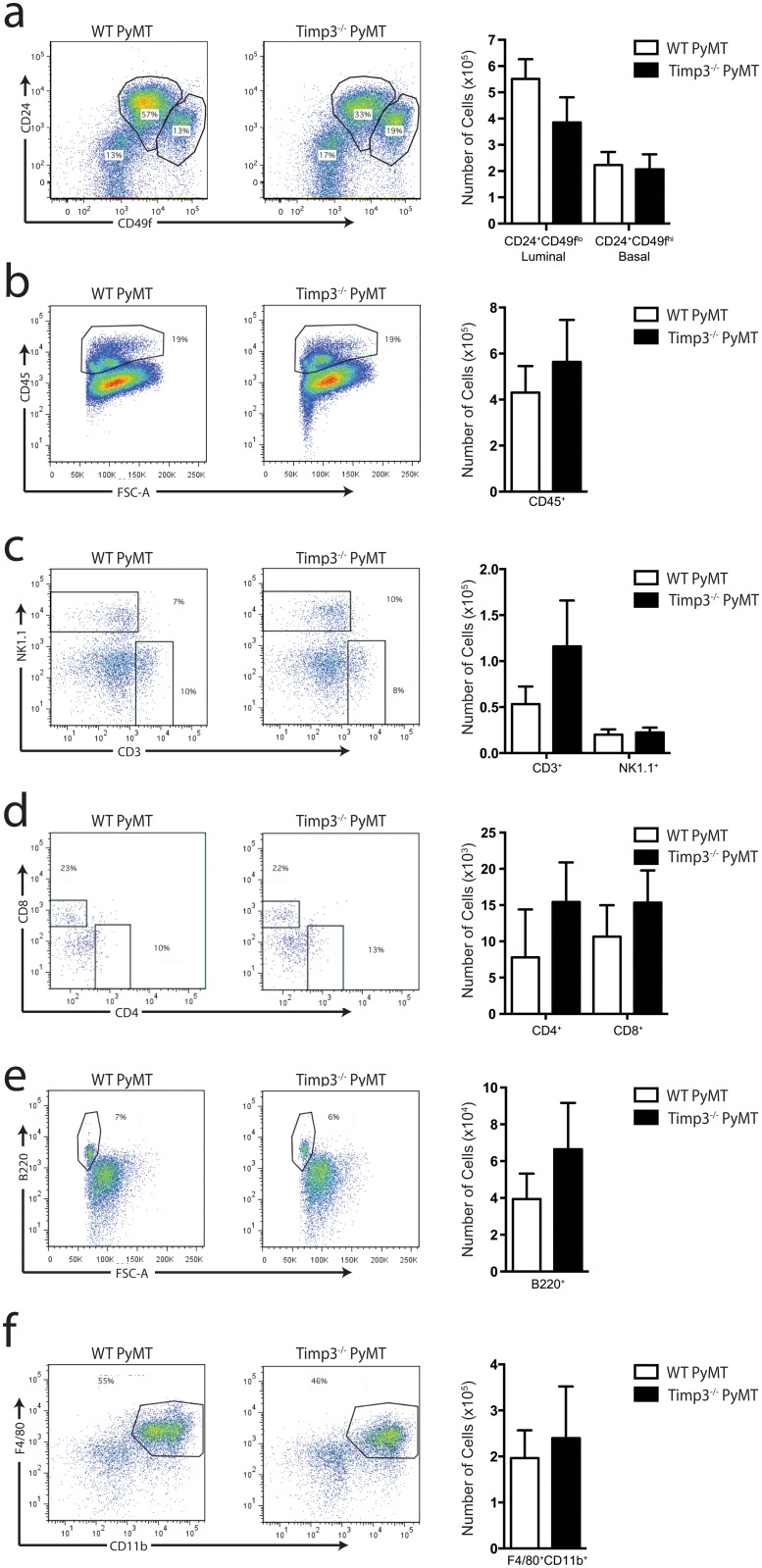
The loss of *Timp3* does not alter the mammary gland immune compartment. Representative flow plots and quantification of (**a**) epithelial luminal and basal populations, (**b**) CD45+ immune cells, (**c**) NK and T cells, (**d**) CD4^+^ and CD8^+^ T cell subsets, (**e**) B cells, and (**f**) macrophages; n = 7, mean ± s.e.m.

The TIMP3-TNF axis is linked to the immune cell response of several tissues including the mammary gland. To investigate immune cell composition and activation during breast cancer onset, MMTV-PyMT mammary glands were dissociated and stained with multiple cell surface markers for flow cytometry quantification using the gating strategy depicted in S2 Fig We compared resident/influxing immune cells, endothelial cells, and epithelial cells in MMTV-PyMT *Timp3* ^*+/+*^ and *Timp3* ^*−⁄−*^ mice during the early window of mammary cancer suppression (day 40). As anticipated, there was a trend towards increased luminal cells in the *Timp3* ^*+/+*^ glands (Fig 4a), a likely indication of the early luminal cell growth and hyperplasia identified histologically (Fig 2a and 2b). Total CD45^+^ immune cells in the mammary gland were unaltered in *PyMT Timp3* ^*−⁄−*^ (Fig 4b). Within this local immune cell population we observed no differences in NK cells (NK1.1^+^, Fig 4c), T cells (CD3^+^, CD4^+^, CD8^+^ Fig. 4c and 4d), B cells (B220^+^, Fig 4e), macrophages (F4/80^+^CD11b^+^, Fig 4f), or in their activation markers (CD69^+^, MHCII^+^CD80^+^, S3 Fig). We also did not find any alterations in endothelial cell numbers (data not shown). Similarly, the immune cell populations of the local lymph node were not altered (S4 Fig) and no apparent differences were noted in the systemic immune cell populations of the mesenteric lymph nodes or spleen (data not shown). Therefore the early suppression of mammary cancer that occurs in the absence of *Timp3*, and requires *Tnfr1*, does not involve the immune cell compartment.

### *Timp3* deficiency in the host delays tumor progression

**Fig 5:**
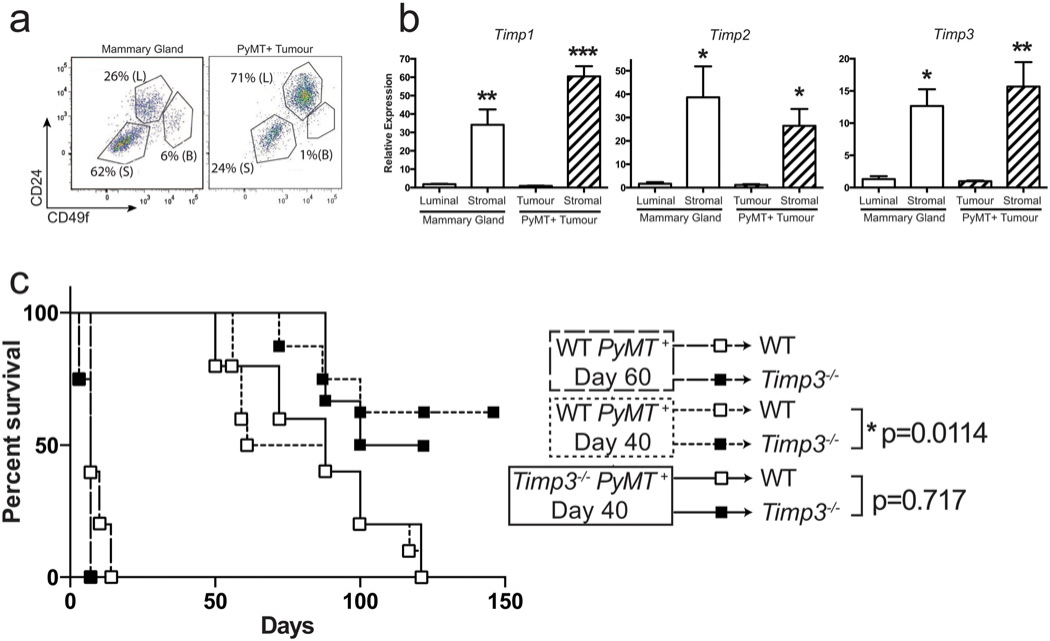
*Timp3* deficiency in the host delays tumor progression. **a**) Representative flow plots of sorted luminal, basal and stromal mammary populations. **b**) RT-PCR of *Timp* gene expression in sorted populations. **c**) Kaplan-Meier curve of the age at first detection of transplanted MMTV-PyMT positive Day 40 (straight) and Day 60 (dotted) *Timp3* ^*+/+*^ cells into *Timp3* ^*+/+*^ (white) and *Timp3* ^*-/-*^ (black) hosts.

TIMP3 is secreted and found localized to the extracellular matrix[20]. Previous work has shown that *Timp3* deficiency in the host, not the tumor, alters the growth of implanted melanoma cells[21]. To identify tissue compartments that express *Timp* genes in the breast and in breast cancer we used fluorescence-activated cell sorting (FACS) to sort different cell types from mammary glands with and without the MMTV-PyMT transgene (Fig 5a). We quantified gene expression of the 4 *Timp* genes in tumor and stromal cell populations as well as the comparable luminal and stromal cells of a healthy PyMT-negative mammary gland. *Timp4* was not detected, but the other 3 *Timp*s were expressed at the highest levels in the CD45^−^CD31^−^ stromal population (Fig 5b).

Next, to elucidate the involvement of the stromal microenvironment in the mammary tumor suppression found in the absence of TIMP3, PyMT cells were isolated and orthotopically transplanted into the mammary fat pads of either *Timp3* ^*+/+*^ control or *Timp3* ^*−⁄−*^ experimental hosts. When cells from day 60 carcinomas were injected, tumors developed quickly in both control and experimental hosts (Fig 5c). In contrast, when PyMT day 40 cells were transplanted, tumors became palpable over a span of 50–120 days in *Timp3* ^*+/+*^ control recipients, but the same tumor cells showed a delay in growth upon transplantation in *Timp3* deficient hosts (Fig 5c). This was true for both *PyMT Timp3* ^*+/+*^ (p = 0.0114) and *PyMT Timp3* ^*−⁄−*^ donor cells (p = 0.0717), and importantly, 50–60% of these *Timp3* ^*−⁄−*^ experimental recipients remained tumor free at 120 days. Interestingly, *Timp3* deficient stroma was able to suppress the growth of early stage tumors with and without *Timp3* (day 40), but not more advanced tumors (WT day 60). Likely, *Timp3* stromal deficiency stalls tumor progression at an early stage in *PyMT Timp3* ^*−⁄−*^ mice resulting in the large differences observed compare to *PyMT Timp3* ^*+/+*^ at 60 days of age (Fig 2). Thus, the loss of *Timp3* from the host stroma specifically inhibits the early steps of tumor progression in spontaneous breast cancer models of *Timp3* null mice.

## Discussion

Here we have described the unexpected impact of *Timp3* in mouse models of human breast cancer. Two independent models show that the loss of *Timp3* results in mammary tumor suppression. Further, *Timp3* deficiency specifically within mammary stroma is sufficient for delayed tumor onset, although the loss of *Timp3* does not alter the mammary immune composition. Through our investigation of the TNF-TIMP3 axis we have also detailed the impact of both TNFR1-specific and total TNF signaling on breast cancer progression. The loss of *Timp3* impacts tumorigenesis akin to *Tnf* or *Tnfr1* deficiency, but requires the presence of *Tnfr1*.

*Timp3* is a common target of methylation or miRNA regulation and is thereby silenced during tumorigenesis[1,22–25]. Other studies have investigated the impact of *Timp3* re-expression or overexpression using fully transformed cell lines[6,26–30], and its expression in advanced human tumors[1,2,31]. Our work utilizes a genetic approach to determine the impact of *Timp3* loss in epithelial and stromal compartments as well as during mammary tumor initiation and progression. The study of *Timp3* expression in human tumor samples has led to the general conclusion that its loss is correlated with advanced tumor stage and even nodal involvement[3,32]. This applies across all breast cancer subtypes, and decreased *Timp3* expression is observed in the more aggressive, hormone receptor negative subset[32] and is correlated with therapeutic success[33]. This data is in line with the aggressive late stage tumorigenesis observed here in mammary cancer of *Timp3* ^*-/-*^ mice and our previous report on melanoma cell lines[21]. Furthermore, our study defines a new role for *Timp3* in the initiation of tumorigenesis. It may be that *Timp3* loss in human cancer is a late event and not an initiating mutation.

Other groups have shown that the overexpression of *Timp3* can protect against tumorigenesis by stabilizing TNF family death receptors on the surface of tumor cells via the blocking of receptor shedding and increasing their sensitivity to extracellular death signals[6,26,29]. In the current study, tumors exhibit only minimal apoptosis, which is unlikely to have altered breast cancer development.

Given the requirement for *Tnfr1* in *Timp3* regulation of breast cancer it is possible that the increased shedding of *Tnfr1* from mammary epithelial cells participates during suppression. This shedding event is known to define the threshold for TNF signaling and maintains an important balance for immune cell function[34]. Increased shedding would directly decrease TNFR1 specific signaling, and would also dampen total TNF signaling since shed TNFR1 complexes with soluble TNF, thereby competing with intact signaling-competent receptors. TNFR1 on mammary tumor cells is known to induce proliferation[35] and we observed that proliferation is dampened in the absence of *Timp3* (Fig 2e). In addition, the role of TNFR2 and the impact of membrane versus soluble TNF remain unknown and both could impact TNF signaling in this model. Future work defining the regulation of TNF signaling during breast cancer progression will be important to target useful therapeutic interventions against this pathway.

The complex role of *Timp3* appears to be similar to the “double-edged sword” of TNF signaling in cancer[36]. TNF has both inducing and protective roles during cancer development. Many of these are related to local and systemic inflammation, a function that is regulated by *Timp3*[10]. Here we conclude that *Timp3*, *Tnf* and *Tnfr1* similarly promote breast cancer onset with varying effects on tumor progression. Even though *Timp3* does not regulate immune cell influx during breast cancer onset, stromal *Timp3* may influence local inflammation. *In vivo*, Sangaletti *et al* have shown that immune cells are an important source of TNF in a model of Neu-induced breast cancer, but its receptor, TNFR1, does not function through the immune compartment[37]. The loss of immune cell *Tnf* caused delayed tumor progression and vascular hemorrhaging[37,38] similar to our observation in endpoint Neu *Timp3* ^*−⁄−*^ tumors (S1 Fig). The regulation of TNF signaling by TIMP3 may comprise an important step in tumor development as revealed in both PyMT and Neu models of breast cancer. Our study underscores the importance of *Timp3* in the mammary gland revealing unexpected but important influences on breast cancer development.

## Methods

### Mouse Strains

All mice used in this study were on an FVB or C57BL/6 background or a combination thereof. *Timp1* ^*−⁄−*38^, *Timp2* ^*−⁄−*39^, *Timp3* ^*−⁄−*40^, *Timp4* ^*−⁄−*41^ mice have been described previously; *Tnf* ^*−⁄−*^(*Tnf* ^*tm1Gkl*^), *Tnfr1* ^*−⁄−*^(*Tnfrsf1a* ^*tm1Mak*^), and *MMTV-PyMT* ^42^(*MMTV-PyVT* 634Mul/J) mice were purchased from the Jackson laboratories. These strains were crossed to generate individual or compound knockouts in the presence of the MMTV-PyMT transgene. Due to differences in mammary gland morphology and timelines of tumor development between these two backgrounds all experiments were compared to matched WildType F2 or F3 generation mice. The MMTV-PyMT transgene was carried by male mice in all breeding pairs. *PyMT Timp1* ^*−⁄−*^, *PyMT Timp3* ^*−⁄−*^, *and PyMT Timp1* ^*−⁄−*^ *Timp2* ^*−⁄−*^ *Timp4* ^*−⁄−*^ mice in Figs 1c, 2e, 2f and 4, and S2–S4 Figs were on a pure C57BL/6 background, while all other crosses were from a FVB or C57BL/6 mixed strain, with MMTV-PyMT originating from FVB. For transplantation experiments only mice from a pure C57BL/6 background were used. All mice were cared for according to guidelines established by the Canadian Council for Animal Care under protocols approved by the Animal Care Committee of the Ontario Cancer Institute (Animal Use Protocol #812). All efforts were made to minimize suffering.

### Tumor parameters

MMTV-PyMT positive female mice were palpated for tumors twice a week starting at 6 weeks of age when on a mixed FVB, C57BL/6 background and at 7 weeks of age when on a pure C57BL/6 background. Tumor onset was measured upon detection of any palpable tumor mass. Tumor size was measured by calliper and tumor endpoint was defined as a mouse having any one tumor of 15mm. Mice were monitored biweekly and sacrificed by CO_2_ at tumor endpoint. Kaplan-meier survival curves and the log-rank statistical test were used to determine differences in tumor onset and overall survival. At tumor endpoint all mammary glands were dissected and weighed. Tumor burden was measured as the ratio of the weight of all tumors compared to the animal’s total body weight. Tumor multiplicity was defined as the total number of glands that had externally visual tumors greater than 5mm in size. At tumor endpoint or unique timepoints (Day 40, Day 60, Day 80) tumors were fixed in 4% PFA or frozen in liguid N_2_ for further analysis.

For the quantification of macroscopic metastatic lesions lungs were fixed and stained in Bouin’s Fixative and lesions were counted using a dissecting scope. For microscopic lesions, all lung lobes were dissected and fixed in 4% PFA overnight at 4°C. One section was made across all lobes and was stained with H&E. Metastatic lesions were scored for one entire lung section per mouse.

For tumor transplant experiments *PyMT Timp3* ^*+/+*^ tumors were dissected at specific time points of 40 or 60 days of age and were dissociated to single cells as previously described[39]. 50,000 total cells were resuspended in a 50% Matrigel, 45% DMEM:F12 media, 5% 0.04% trypan blue mixture before being injected orthotopically into the mammary fat pads of isofluorane/oxygen anaesthetized adult host mice. Recipient glands were palpated twice weekly beginning a week after injection.

### Tissue Fixation and Staining

Wholemount and H&E staining was performed as previously described[39].

For flow cytometry staining mammary glands were dissociated by collagenase digestion as previously described in Joshi et al and lymph nodes and spleens were collected and stained as described in Murthy et al with the addition of anti-MHCII (clone M5/114.15.2), anti-CD80 (clone 16-10A1), and anti-CD45.2 (clone 104)[39,40].

### Statistical Analysis

Statistical significance was determined using student’s T-test or ANOVA with Tukey’s multiple comparisons test for all histograms and Kaplan-Meier survival curves were analyzed using a Mantel-Cox Log-rank test. Statistical significance was depicted as *p<0.05, **p<0.01, ***p<0.001.

## Supporting Information

S1 Fig First palpation and tumor endpoint. **a**) Histogram of the number of days to first palpation and from first palpation to tumor endpoint in FVB MMTV-PyMT mice (mean ± s.e.m. *Timp3* ^*+⁄+*^ n = 25, *Timp3* ^*+⁄−*^ n = 17, *Timp3* ^*−⁄−*^ n = 21). **b**) Histogram of the number of days to first palpation and from first palpation to tumor endpoint in FVB MMTV-Neu mice (mean ± s.e.m. *Timp3* ^*+⁄+*^ n = 21, *Timp3* ^*+⁄−*^ n = 20, *Timp3* ^*−⁄−*^ n = 22).
(EPS)

S2 Fig Gating strategy for mammary gland immune cell analysis. Debris and doublets were removed using forward scatter and side scatter area, height and width parameters. Dead cells were then removed using the viability dye DAPI. Next, total immune cells were identified by their expression of the pan-immune surface marker CD45.2 and within this subset specific immune cell populations were identified. T cells were categorized as positive for CD3 expression and negative for NK1.1 expression and further categorization of T cells was completed using CD4 and CD8, as well as CD69 (a marker of lymphocyte activation). B cells were identified as having positive B220 surface expression. Finally, macrophages were identified by their positive expression of surface markers CD11b and F4/80, and further characterized by their activation markers CD80 and MHCII.
(EPS)

S3 Fig Quantification of mammary immune cell activation. Representative flow plots and quantification of (**a**) CD69^+^ CD4^+^ T cells, (**b**) CD69^+^ CD8^+^ T cells, and (**c**) CD80^+^MHCII^+^ Macrophages.
(EPS)

S4 Fig Quantification of mammary lymph node immune cells. Representative flow plots and quantification of (**a**) CD45+ immune cells, (**b**) T cells, (**c**) B cells, and (**d**) macrophages.
(EPS)
